# Subcapsular haematoma following laparoscopic cholecystectomy

**DOI:** 10.1259/bjrcr.20160118

**Published:** 2016-12-23

**Authors:** Brian M Moloney, Niamh Hennessy, Eoin O Malley, Felix Orefuwa, Peter A McCarthy, Chris G Collins

**Affiliations:** ^1^Department of Surgery, Portiuncula University Hospital, Saolta University Healthcare Group, Galway, Ireland; ^2^Department of Surgery, Galway University Hospital, Saolta University Healthcare Group, Galway, Ireland; ^3^Department of Radiology, Galway University Hospital, Saolta University Healthcare Group, Galway, Ireland

## Abstract

Laparoscopic cholecystectomy (LC) is now considered the gold standard treatment for symptomatic gallbladder disease. Over the last two decades, a reduction in postoperative morbidity, mortality and hospital stay have seen a complete shift from open surgery to a laparoscopic approach. Intrahepatic subcapsular haematoma (ISH) is a rare and potentially life-threatening complication of LC. A 34-year-old female underwent LC for uncomplicated cholelithiasis. No complications were observed intra-operatively. 2 h postoperatively, the patient developed severe abdominal pain and tachycardia. Ultrasonography demonstrated an echogenic collection adjacent to the gallbladder fossa. Laparoscopy showed an ISH involving the right and left lobes of the liver, and no evidence of any intra-abdominal haemorrhage. Subsequent urgent triphasic CT identified a large ISH and a hypervascular lesion on the right lobe of the liver. This lesion demonstrated delayed enhancement with contrast filling suggestive of a hepatic haemangioma. This case report demonstrates the impact of imaging on postoperative management and the importance of postoperative patient monitoring in patients who have undergone laparoscopic surgery. Imaging explorations have a decisive role in the detection and characterization of haematomas.

## Clinical presentation

A 34-year-old female presented electively for laparoscopic cholecystectomy (LC) for uncomplicated cholelithiasis. No other significant past medical history was documented. Ultrasound imaging prior to surgery showed cholelithiasis without signs of cholecystitis. Preoperative blood tests were normal. The patient received perioperative enoxaparin (40 mg) as venous thromboembolic prophylaxis. She underwent LC using four trocars: one 10-mm trocar and three 5-mm trocars. The dissection of the gallbladder from the liver bed was accomplished without concern, and the gallbladder was removed intact through the periumbilical port without evidence of rupture. LC was performed without complication. The duration of the procedure was 45 min. Within 2 h following the procedure, she developed severe right shoulder and upper abdominal pain. On examination, the patient was tachycardic (114 bpm) and her blood pressure was 128/60 mmHg; upper abdominal tenderness was elicited on abdominal palpation. Blood tests revealed an acute reduction in haemoglobin level from 13.4 g dl^–1^ (preoperatively) to 9.9 g dl^–1^ (3 h post-procedure).

## Differential diagnoses

- Haemorrhage
  - Intra-abdominal vessel injury
    - Vena cava
    - Portal vein
    - Cystic artery
    - Hepatic artery
  - Bleeding from the liver bed
  - Bleeding from the vessels of the abdominal wall

## Investigation/imaging findings

**Figure 1.**
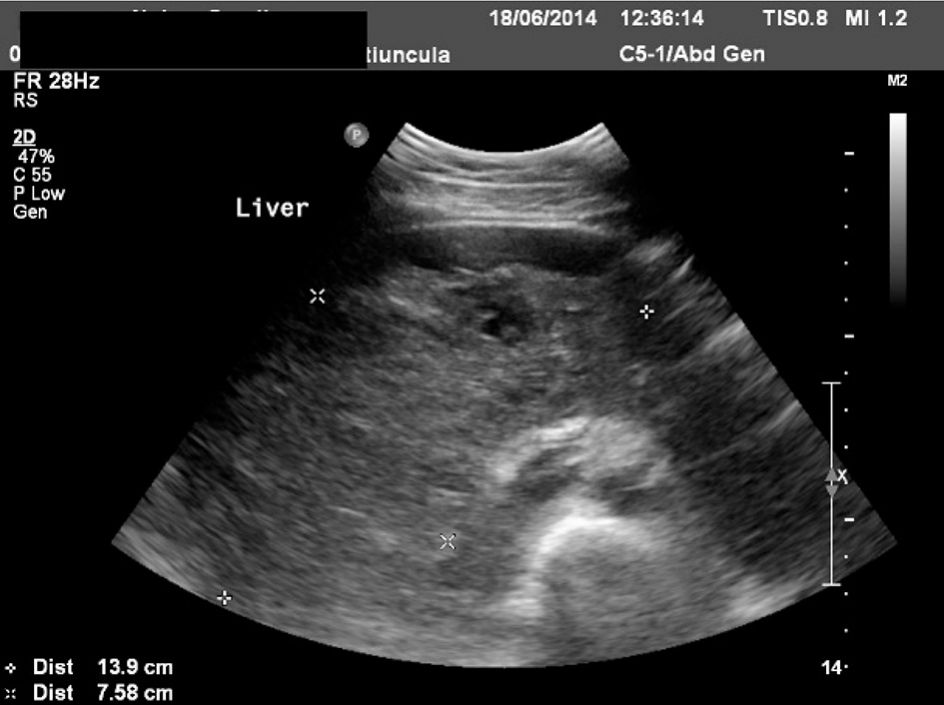
Ultrasonography demonstrated an echogenic collection in gallbladder fossa measuring 13 × 7 cm.

**Figure 2.**
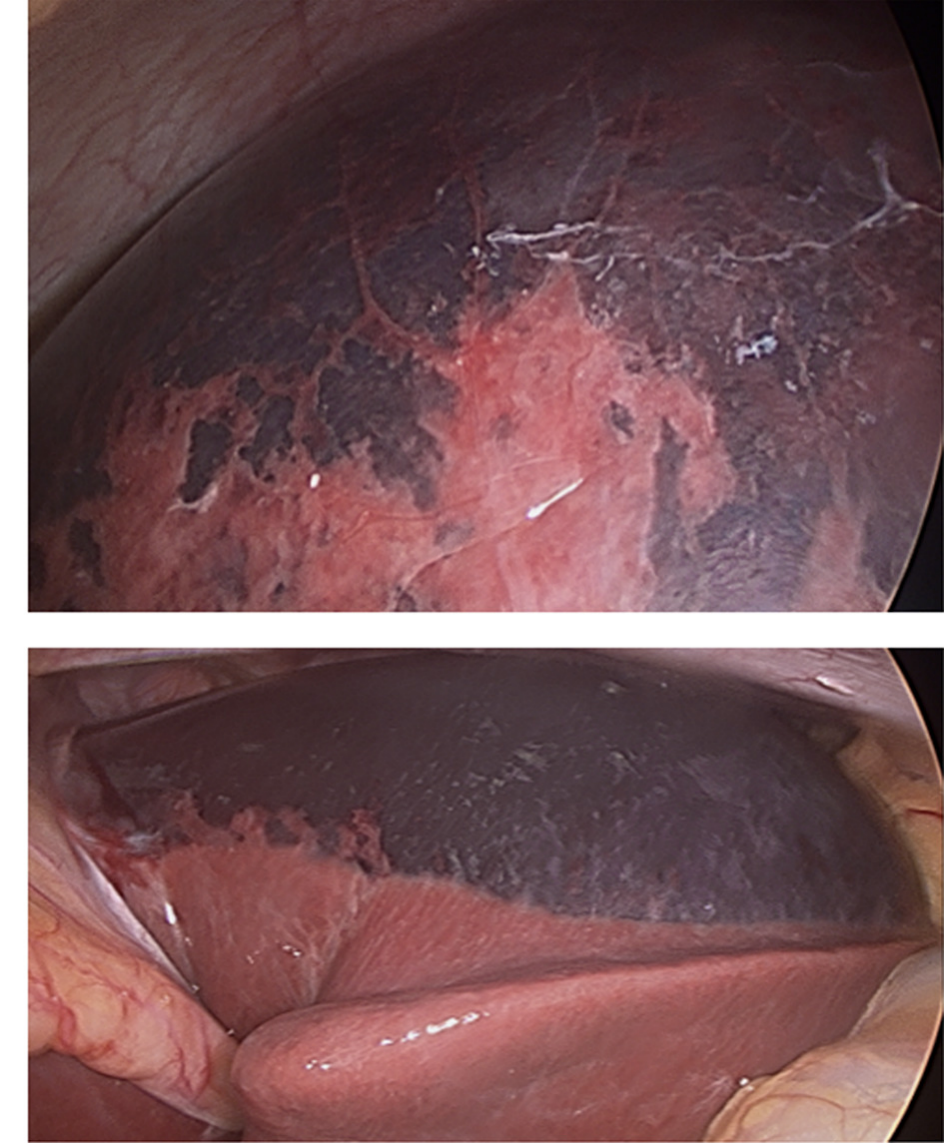
Laparoscopy identified a large intracapsular hepatic haematoma involving the right and left lobes of the liver.

**Figure 3.**
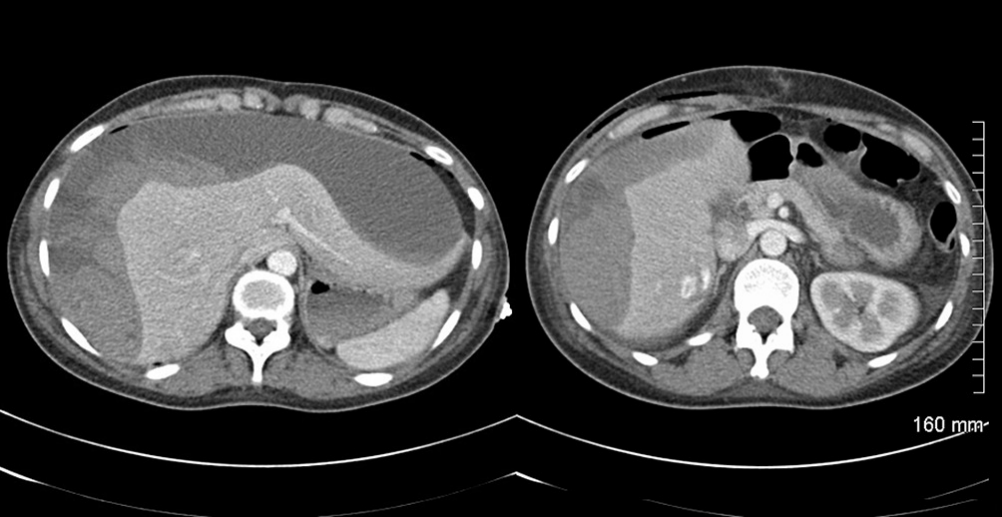
Subsequent triphasic CT identified a large subcapsular liver haematoma (left) and a hypervascular lesion on the right lobe of the liver (right).

**Figure 4.**
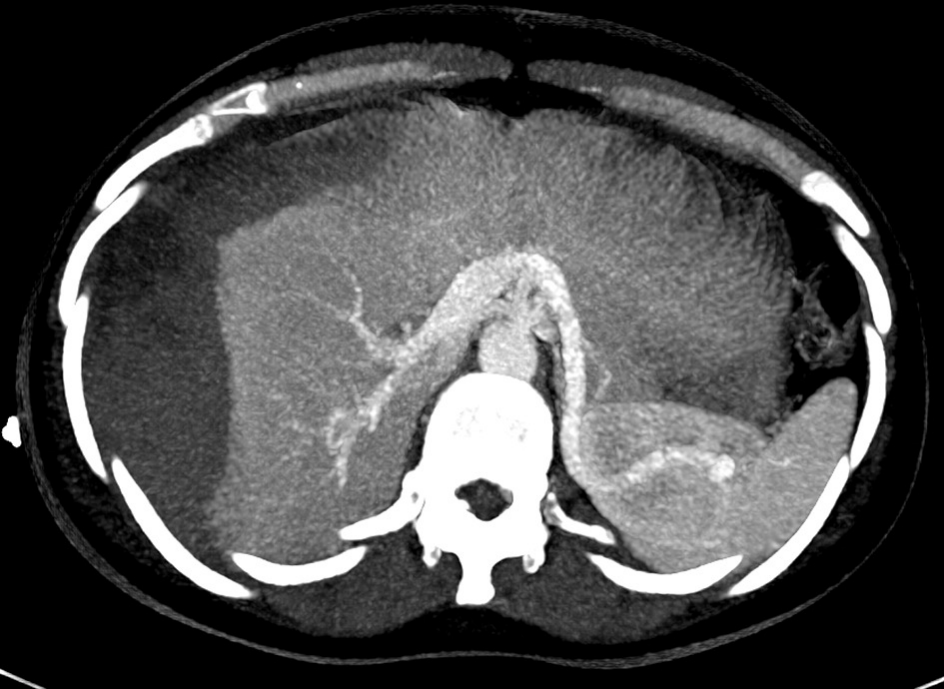
Portal venous phase axial maximum intensity projection (MIP) image demonstrating intact vasculature with no visible bleeding from the haemangioma.

**Figure 5.**
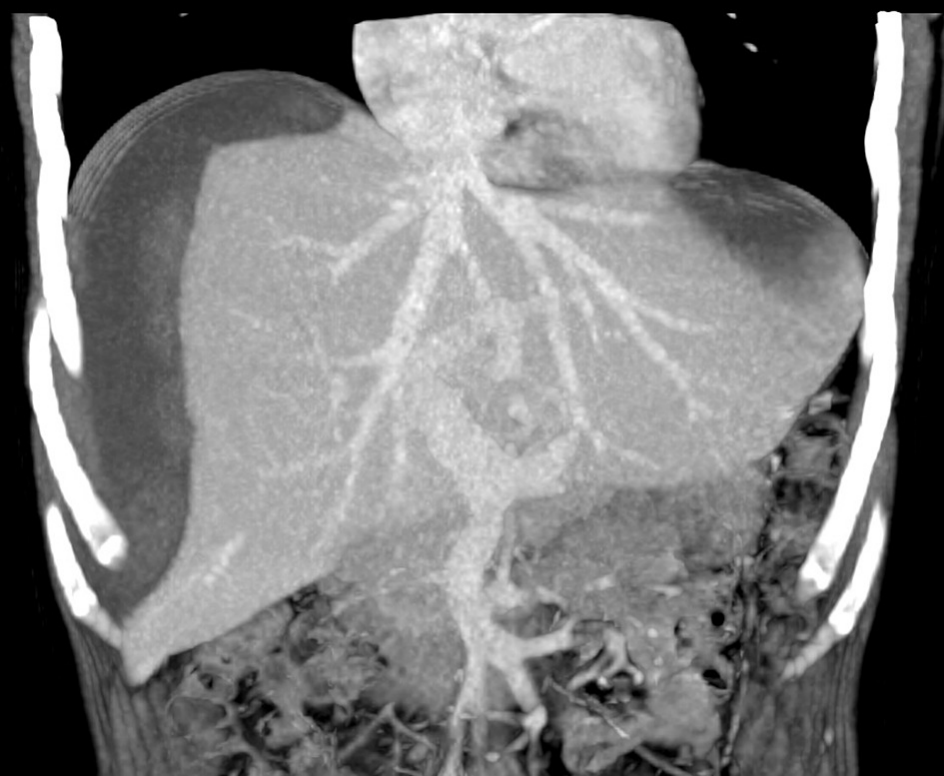
Portal venous phase coronal maximum intensity projection (MIP) image detailing vessel integrity.

Abdominal ultrasonography revealed an echogenic localized collection in the gallbladder fossa measuring 13 × 7 cm (Figure 1). The patient underwent an emergency laparoscopy. Intra-operatively, a large intrahepatic subcapsular haematoma (ISH) involving the right and left lobes of the liver was identified (Figure 2). There was no evidence of bleeding from the gallbladder bed and there was no evidence of any intra-abdominal haemorrhage. Postoperatively, an urgent triphasic CT of the liver was performed. A large ISH, with marked compression of the liver, and a hypervascular lesion over the posterior aspect of the right lobe of the liver were identified (Figure 3). This lesion demonstrated delayed enhancement with contrast filling, suggestive of a hepatic haemangioma. No hepatic arterial, portal venous or hepatic venous compromise was visualized (Figures 4 and 5).

## Treatment

The patient was managed conservatively with close monitoring of the patient’s clinical status and haemoglobin level. Transfusion was not deemed necessary as the haemoglobin was maintained over 8 g/dl^–1^. Postoperatively, this patient reported a history of recurrent epistaxis and easy bruising; however, she did not report a history of menorrhagia. She had three normal vaginal deliveries and denied any post-partum haemorrhage. There was no family history of a bleeding disorder and a personal history was investigated and excluded.

The patient was observed over 4 days with daily blood tests, and was discharged with stable haemoglobin levels. Following discharge, the patient was re-admitted twice within 2 weeks of initial surgery for further pain management. Ultrasound examination of the liver showed persistence of the ISH, and CT abdomen showed interval decrease in the size of the ISH.

## Discussion

LC is now globally accepted as the mainstay treatment of choice for symptomatic gallbladder disease.^1^ In recent years, LC has been acknowledged as a possible procedure for day case surgery, in the suitable patient, owing to advancements in operative and anaesthetic techniques, together with increased familiarity with the procedure.^1,2^ Although considered as safe a procedure as open cholecystectomy,^3^ surgical complications are well documented. Serious complications associated with LC occur in 2.6% of cases.^4^ These complications include haemorrhage, gallbladder perforation, common bile duct injuries and conversion to open surgery intra-operatively, and haemorrhage, subhepatic abscess, bile leak and choledocholithiasis postoperatively.^5^ The occurrence of postoperative haemorrhage is rare (0.08–2%).^3,6^ ISH, although very uncommon, is among the most severe complications. Common bleeding sites include the gallbladder fossa, the abdominal trocar insertion site, the cystic artery, the falciform ligament and bleeding from a liver capsule tear. Specifically, ISH of the liver without intraabdominal haemorrhage is an extremely rare and potentially life-threatening complication of LC.

In this case, the patient presented with sudden-onset severe right upper quadrant pain and tenderness, she was tachycardic and laboratory investigations showed an acute decrease in haemoglobin. A similar presentation of acute right-sided abdominal pain has been documented in other cases of ISH.^7^ Patients may also present with haemodynamic shock if rupture of the liver capsule has occurred with intraabdominal bleeding. Furthermore, an infected haematoma may occur whereby the patient may present with pyrexia, abdominal pain and features of sepsis. Bacterial translocation from the gut is thought to be the cause of this superimposed infection.^8^

Given the findings on CT, the author postulates that the cause of ISH was spontaneous haemorrhage of liver parenchyma following normal manipulation of the gallbladder and liver intraoperatively. Of note, however, a hepatic haemangioma is visible adjacent to Glisson’s capsule (Figures 3 and 4). Haemangiomas are the most common benign liver tumours with an estimated prevalence of 5–20%.^9^ Generally indolent, many symptomatic patients have the tumour identified by radiological imaging tests of the abdomen for other reasons. During LC, the lower hepatic surface is put under significant traction to adequately expose the operating field to the surgeon. This traction may be adequate to cause haemorrhage of a vulnerable haemangioma or surrounding liver parenchyma. While a high level of suspicion of haemorrhage is warranted considering the vascularity of haemangiomas, in this case, maximum intensity projection (MIP) imaging demarcates the vessel integrity around this lesion and renders it less likely that the hepatic haemangioma was the specific source of the haemorrhage. Despite a thorough review of imaging in multiple planes and using MIP imaging, a site of haemorrhage could not be clearly demarcated.

A known history of haemangiomas should be considered pre, intra and postoperatively. Preoperatively, anticoagulation need should be determined and omitted if unnecessary. Also, further imaging such as CT scanning and/or MRI could be considered to characterize a haemangioma. Intraoperatively, liver traction should be minimized through careful trocar placement while still allowing a critical view of safety to prevent visceral injury. Postoperatively, a high index of suspicion and early imaging can detect haemorrhage, facilitating an expedited treatment.

Other possible causes of ISH following LC due to surgical variation have been documented. These include damage due to trocar placement,^10^ excessive traction of the gallbladder causing injury to the liver capsule^8^ and excessive manipulation of the liver during dissection of the gallbladder.^11^ Ketorolac, a non-steroidal anti-inflammatory injection used for perioperative pain management, has been associated with ISH in a number of cases.^6,12,13^ Complications associated to ketorolac are not limited to ISH, with as many as 97 reactions with a fatal outcome between 1990 to 1993 resulting in the drug being withdrawn from many countries.^14^ Anatomical variations such as the presence of a pseudoaneurysm are considered a risk factor. One case describes the development of a subcapsular haematoma 1 week after cholecystectomy by open approach. Selective angiography identified the presence of pseudoaneurysm in the right hepatic artery.

The management of ISH is dependent on the patient’s clinical status and the size of the haematoma. In this case, the patient was considered to be clinically unstable, showing signs of active bleeding such as persistent severe abdominal pain, tachycardia, and an acute reduction in haemoglobin levels. As such, an emergent diagnostic laparoscopy was performed. If the patient is stable and asymptomatic, a conservative approach may be taken. This can be considered if the haematoma is confined to the capsule with no intraabdominal haemorrhage, and the haematoma is small.^7^ A conservative approach involves close observation of the haematoma. If the haematoma is large and unlikely reabsorbed conservatively, an ultrasound-guided percutaneous drain can be placed.^7^ In particular, if the patient develops signs of sepsis/an infected haematoma, radiological drainage of the haematoma can be considered,^15^ along with antimicrobial administration.^8,15^ One case describes the use of selective embolization in the case of active bleeding, followed by surgical evacuation if this fails.^8^

## Conclusions

To date, there are very few cases of ISH documented in the literature. ISH is an extremely rare complication following LC. This case reiterates the need to consider the potentially life-threatening complications in patients presenting with acute abdominal pain post-LC. Appropriate imaging guided both diagnosis and further management for this patient.

## Learning points

1. This case report provides an overview of how intrahepatic subscapular haematoma presents and how it is appropriately diagnosed and managed.
2. Intrahepatic subcapsular haematoma should be considered in the differential diagnosis of patients complaining of abdominal pain post-LC.
3. Timely and appropriate imaging can be the key to diagnosis.
4. Follow-up interval ultrasonography can help investigate for resolution.

## Consent

Written informed consent for the case to be published (including images, case history and data) was obtained from the patient(s) for publication of this case report, including accompanying images.
